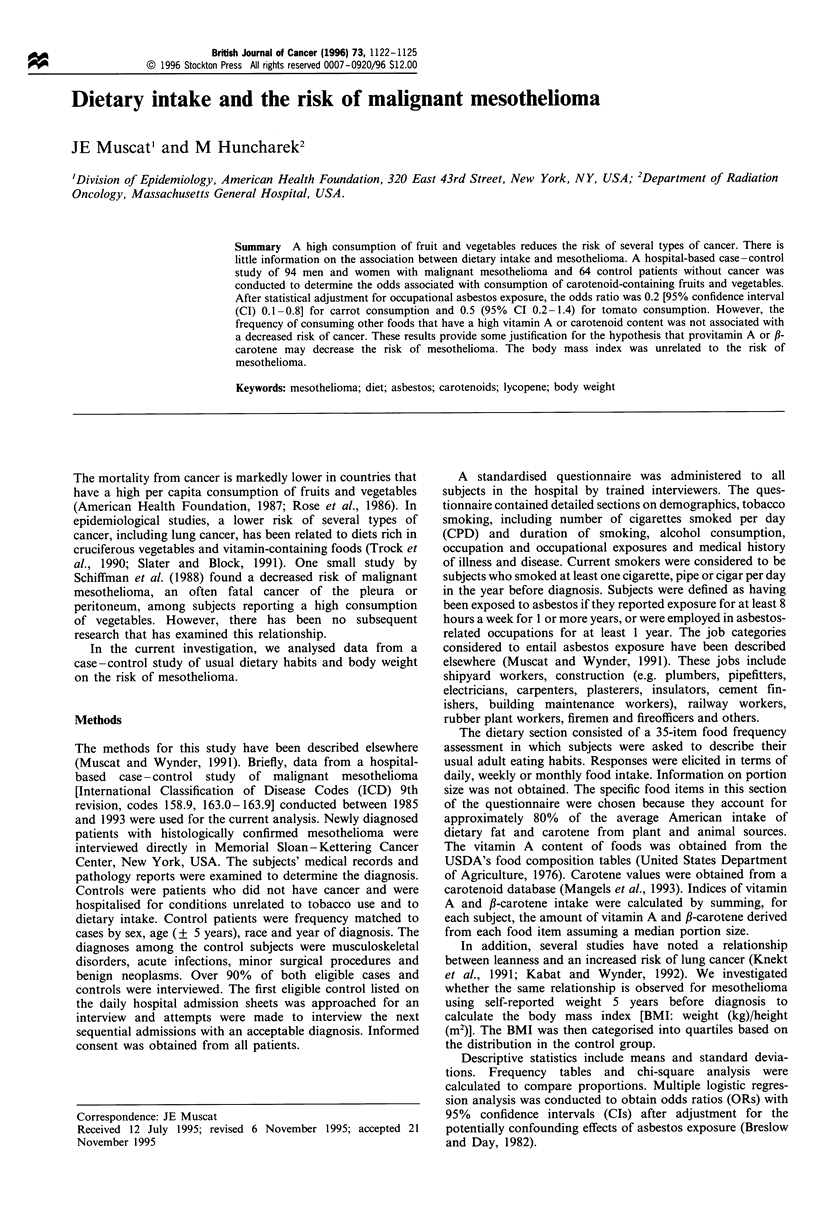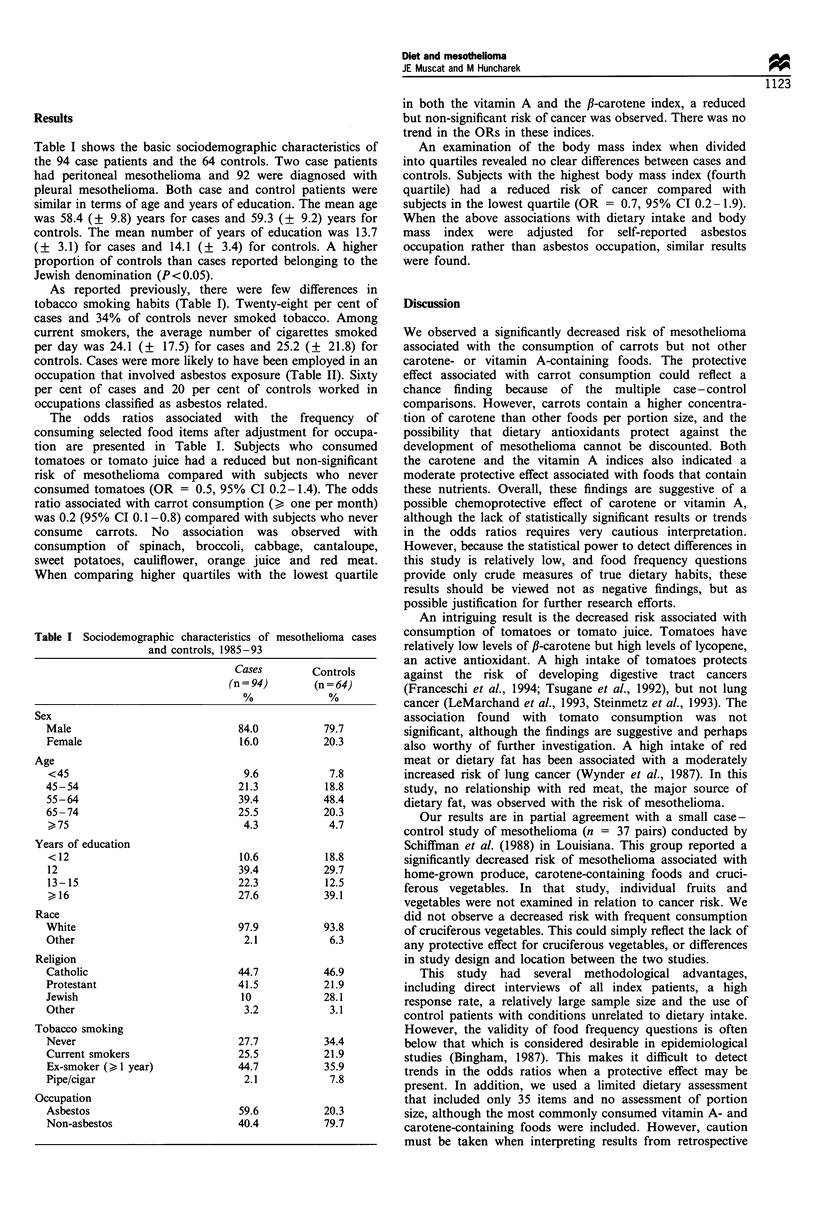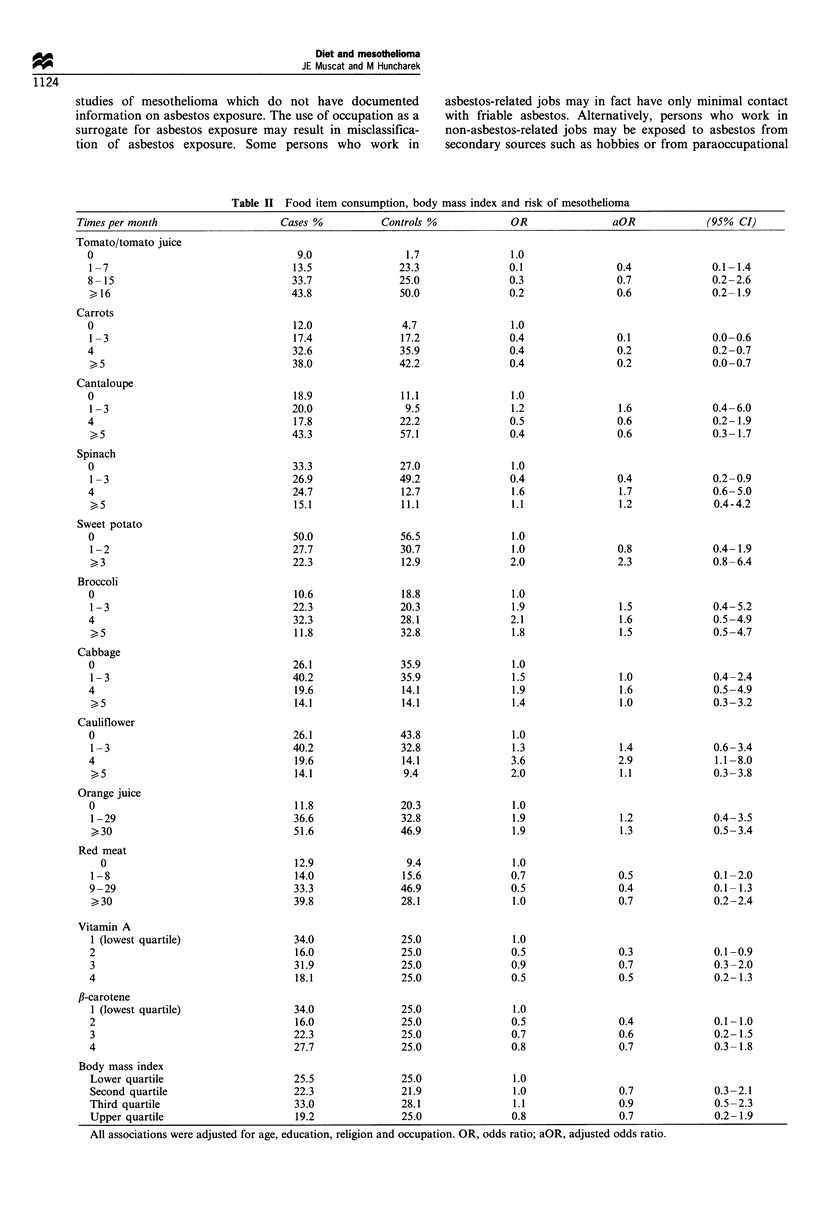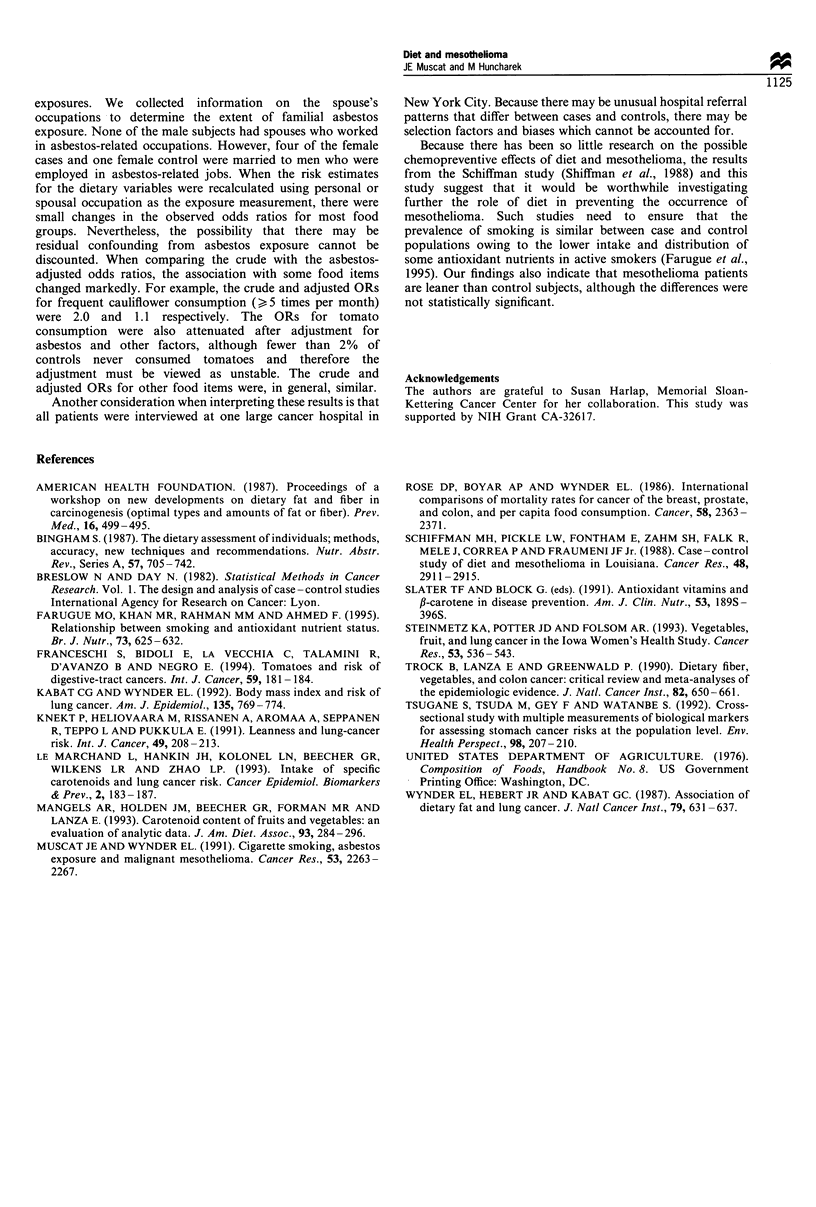# Dietary intake and the risk of malignant mesothelioma.

**DOI:** 10.1038/bjc.1996.215

**Published:** 1996-05

**Authors:** J. E. Muscat, M. Huncharek

**Affiliations:** Division of Epidemiology, American Health Foundation, New York, NY, USA.

## Abstract

A high consumption of fruit and vegetables reduces the risk of several types of cancer. There is little information on the association between dietary intake and mesothelioma. A hospital-based case-control study of 94 men and women with malignant mesothelioma and 64 control patients without cancer was conducted to determine the odds associated with consumption of carotenoid-containing fruits and vegetables. After statistical adjustment for occupational asbestos exposure, the odds ratio was 0.2 [95% confidence interval (CI) 0.1-0.8] for carrot consumption and 0.5 (95% CI 0.2-1.4) for tomato consumption. However, the frequency of consuming other foods that have a high vitamin A or carotenoid content was not associated with a decreased risk of cancer. These results provide some justification for the hypothesis that provitamin A or beta-carotene may decrease the risk of mesothelioma. The body mass index was unrelated to the risk of mesothelioma.


British Journal of Cancer (1996) 73, 1122–1125
© 1996 Stockton Press All rights reserved 0007-0920/96 $12.00

### Dietary intake and the risk of malignant mesothelioma

JE Muscat[1] and M Huncharek[2]

[1]*Division of Epidemiology, American Health Foundation, 320 East 43rd Street, New York, NY, USA;* [2]*Department of Radiation Oncology, Massachusetts General Hospital, USA.*

**Summary** A high consumption of fruit and vegetables reduces the risk of several types of cancer. There is little information on the association between dietary intake and mesothelioma. A hospital-based case–control study of 94 men and women with malignant mesothelioma and 64 control patients without cancer was conducted to determine the odds associated with consumption of carotenoid-containing fruits and vegetables. After statistical adjustment for occupational asbestos exposure, the odds ratio was 0.2 [95% confidence interval (CI) 0.1–0.8] for carrot consumption and 0.5 (95% CI 0.2–1.4) for tomato consumption. However, the frequency of consuming other foods that have a high vitamin A or carotenoid content was not associated with a decreased risk of cancer. These results provide some justification for the hypothesis that provitamin A or $\beta$-carotene may decrease the risk of mesothelioma. The body mass index was unrelated to the risk of mesothelioma.

**Keywords:** mesothelioma; diet; asbestos; carotenoids; lycopene; body weight

The mortality from cancer is markedly lower in countries that have a high per capita consumption of fruits and vegetables (American Health Foundation, 1987; Rose *et al.*, 1986). In epidemiological studies, a lower risk of several types of cancer, including lung cancer, has been related to diets rich in cruciferous vegetables and vitamin-containing foods (Trock *et al.*, 1990; Slater and Block, 1991). One small study by Schiffman *et al.* (1988) found a decreased risk of malignant mesothelioma, an often fatal cancer of the pleura or peritoneum, among subjects reporting a high consumption of vegetables. However, there has been no subsequent research that has examined this relationship.

In the current investigation, we analysed data from a case–control study of usual dietary habits and body weight on the risk of mesothelioma.

### Methods

The methods for this study have been described elsewhere (Muscat and Wynder, 1991). Briefly, data from a hospital-based case–control study of malignant mesothelioma [International Classification of Disease Codes (ICD) 9th revision, codes 158.9, 163.0–163.9] conducted between 1985 and 1993 were used for the current analysis. Newly diagnosed patients with histologically confirmed mesothelioma were interviewed directly in Memorial Sloan–Kettering Cancer Center, New York, USA. The subjects' medical records and pathology reports were examined to determine the diagnosis. Controls were patients who did not have cancer and were hospitalised for conditions unrelated to tobacco use and to dietary intake. Control patients were frequency matched to cases by sex, age (± 5 years), race and year of diagnosis. The diagnoses among the control subjects were musculoskeletal disorders, acute infections, minor surgical procedures and benign neoplasms. Over 90% of both eligible cases and controls were interviewed. The first eligible control listed on the daily hospital admission sheets was approached for an interview and attempts were made to interview the next sequential admissions with an acceptable diagnosis. Informed consent was obtained from all patients.

Correspondence: JE Muscat
Received 12 July 1995; revised 6 November 1995; accepted 21 November 1995

A standardised questionnaire was administered to all subjects in the hospital by trained interviewers. The questionnaire contained detailed sections on demographics, tobacco smoking, including number of cigarettes smoked per day (CPD) and duration of smoking, alcohol consumption, occupation and occupational exposures and medical history of illness and disease. Current smokers were considered to be subjects who smoked at least one cigarette, pipe or cigar per day in the year before diagnosis. Subjects were defined as having been exposed to asbestos if they reported exposure for at least 8 hours a week for 1 or more years, or were employed in asbestos-related occupations for at least 1 year. The job categories considered to entail asbestos exposure have been described elsewhere (Muscat and Wynder, 1991). These jobs include shipyard workers, construction (e.g. plumbers, pipefitters, electricians, carpenters, plasterers, insulators, cement finishers, building maintenance workers), railway workers, rubber plant workers, firemen and fireofficers and others.

The dietary section consisted of a 35-item food frequency assessment in which subjects were asked to describe their usual adult eating habits. Responses were elicited in terms of daily, weekly or monthly food intake. Information on portion size was not obtained. The specific food items in this section of the questionnaire were chosen because they account for approximately 80% of the average American intake of dietary fat and carotene from plant and animal sources. The vitamin A content of foods was obtained from the USDA's food composition tables (United States Department of Agriculture, 1976). Carotene values were obtained from a carotenoid database (Mangels *et al.*, 1993). Indices of vitamin A and $\beta$-carotene intake were calculated by summing, for each subject, the amount of vitamin A and $\beta$-carotene derived from each food item assuming a median portion size.

In addition, several studies have noted a relationship between leanness and an increased risk of lung cancer (Knekt *et al.*, 1991; Kabat and Wynder, 1992). We investigated whether the same relationship is observed for mesothelioma using self-reported weight 5 years before diagnosis to calculate the body mass index [BMI: weight (kg)/height ($m^2$)]. The BMI was then categorised into quartiles based on the distribution in the control group.

Descriptive statistics include means and standard deviations. Frequency tables and chi-square analysis were calculated to compare proportions. Multiple logistic regression analysis was conducted to obtain odds ratios (ORs) with 95% confidence intervals (CIs) after adjustment for the potentially confounding effects of asbestos exposure (Breslow and Day, 1982).

### Results

Table I shows the basic sociodemographic characteristics of the 94 case patients and the 64 controls. Two case patients had peritoneal mesothelioma and 92 were diagnosed with pleural mesothelioma. Both case and control patients were similar in terms of age and years of education. The mean age was 58.4 (± 9.8) years for cases and 59.3 (± 9.2) years for controls. The mean number of years of education was 13.7 (± 3.1) for cases and 14.1 (± 3.4) for controls. A higher proportion of controls than cases reported belonging to the Jewish denomination ($P<0.05$).

As reported previously, there were few differences in tobacco smoking habits (Table I). Twenty-eight per cent of cases and 34% of controls never smoked tobacco. Among current smokers, the average number of cigarettes smoked per day was 24.1 (± 17.5) for cases and 25.2 (± 21.8) for controls. Cases were more likely to have been employed in an occupation that involved asbestos exposure (Table II). Sixty per cent of cases and 20 per cent of controls worked in occupations classified as asbestos related.

The odds ratios associated with the frequency of consuming selected food items after adjustment for occupation are presented in Table I. Subjects who consumed tomatoes or tomato juice had a reduced but non-significant risk of mesothelioma compared with subjects who never consumed tomatoes (OR = 0.5, 95% CI 0.2–1.4). The odds ratio associated with carrot consumption (⩾ one per month) was 0.2 (95% CI 0.1–0.8) compared with subjects who never consume carrots. No association was observed with consumption of spinach, broccoli, cabbage, cantaloupe, sweet potatoes, cauliflower, orange juice and red meat. When comparing higher quartiles with the lowest quartile in both the vitamin A and the β-carotene index, a reduced but non-significant risk of cancer was observed. There was no trend in the ORs in these indices.

An examination of the body mass index when divided into quartiles revealed no clear differences between cases and controls. Subjects with the highest body mass index (fourth quartile) had a reduced risk of cancer compared with subjects in the lowest quartile (OR = 0.7, 95% CI 0.2–1.9). When the above associations with dietary intake and body mass index were adjusted for self-reported asbestos occupation rather than asbestos occupation, similar results were found.

**Table I** Sociodemographic characteristics of mesothelioma cases and controls, 1985–93

| | *Cases* (n=*94*) % | Controls (n=*64*) % |
|---|---|---|
| Sex | | |
| Male | 84.0 | 79.7 |
| Female | 16.0 | 20.3 |
| Age | | |
| <45 | 9.6 | 7.8 |
| 45–54 | 21.3 | 18.8 |
| 55–64 | 39.4 | 48.4 |
| 65–74 | 25.5 | 20.3 |
| ⩾75 | 4.3 | 4.7 |
| Years of education | | |
| <12 | 10.6 | 18.8 |
| 12 | 39.4 | 29.7 |
| 13–15 | 22.3 | 12.5 |
| ⩾16 | 27.6 | 39.1 |
| Race | | |
| White | 97.9 | 93.8 |
| Other | 2.1 | 6.3 |
| Religion | | |
| Catholic | 44.7 | 46.9 |
| Protestant | 41.5 | 21.9 |
| Jewish | 10 | 28.1 |
| Other | 3.2 | 3.1 |
| Tobacco smoking | | |
| Never | 27.7 | 34.4 |
| Current smokers | 25.5 | 21.9 |
| Ex-smoker (⩾1 year) | 44.7 | 35.9 |
| Pipe/cigar | 2.1 | 7.8 |
| Occupation | | |
| Asbestos | 59.6 | 20.3 |
| Non-asbestos | 40.4 | 79.7 |

### Discussion

We observed a significantly decreased risk of mesothelioma associated with the consumption of carrots but not other carotene- or vitamin A-containing foods. The protective effect associated with carrot consumption could reflect a chance finding because of the multiple case–control comparisons. However, carrots contain a higher concentration of carotene than other foods per portion size, and the possibility that dietary antioxidants protect against the development of mesothelioma cannot be discounted. Both the carotene and the vitamin A indices also indicated a moderate protective effect associated with foods that contain these nutrients. Overall, these findings are suggestive of a possible chemoprotective effect of carotene or vitamin A, although the lack of statistically significant results or trends in the odds ratios requires very cautious interpretation. However, because the statistical power to detect differences in this study is relatively low, and food frequency questions provide only crude measures of true dietary habits, these results should be viewed not as negative findings, but as possible justification for further research efforts.

An intriguing result is the decreased risk associated with consumption of tomatoes or tomato juice. Tomatoes have relatively low levels of β-carotene but high levels of lycopene, an active antioxidant. A high intake of tomatoes protects against the risk of developing digestive tract cancers (Franceschi *et al.*, 1994; Tsugane *et al.*, 1992), but not lung cancer (LeMarchand *et al.*, 1993, Steinmetz *et al.*, 1993). The association found with tomato consumption was not significant, although the findings are suggestive and perhaps also worthy of further investigation. A high intake of red meat or dietary fat has been associated with a moderately increased risk of lung cancer (Wynder *et al.*, 1987). In this study, no relationship with red meat, the major source of dietary fat, was observed with the risk of mesothelioma.

Our results are in partial agreement with a small case–control study of mesothelioma ($n$ = 37 pairs) conducted by Schiffman *et al.* (1988) in Louisiana. This group reported a significantly decreased risk of mesothelioma associated with home-grown produce, carotene-containing foods and cruciferous vegetables. In that study, individual fruits and vegetables were not examined in relation to cancer risk. We did not observe a decreased risk with frequent consumption of cruciferous vegetables. This could simply reflect the lack of any protective effect for cruciferous vegetables, or differences in study design and location between the two studies.

This study had several methodological advantages, including direct interviews of all index patients, a high response rate, a relatively large sample size and the use of control patients with conditions unrelated to dietary intake. However, the validity of food frequency questions is often below that which is considered desirable in epidemiological studies (Bingham, 1987). This makes it difficult to detect trends in the odds ratios when a protective effect may be present. In addition, we used a limited dietary assessment that included only 35 items and no assessment of portion size, although the most commonly consumed vitamin A- and carotene-containing foods were included. However, caution must be taken when interpreting results from retrospective studies of mesothelioma which do not have documented information on asbestos exposure. The use of occupation as a surrogate for asbestos exposure may result in misclassification of asbestos exposure. Some persons who work in asbestos-related jobs may in fact have only minimal contact with friable asbestos. Alternatively, persons who work in non-asbestos-related jobs may be exposed to asbestos from secondary sources such as hobbies or from paraoccupational

**Table II** Food item consumption, body mass index and risk of mesothelioma

| *Times per month* | *Cases %* | *Controls %* | *OR* | *aOR* | *(95% CI)* |
|---|---|---|---|---|---|
| Tomato/tomato juice | | | | | |
| 0 | 9.0 | 1.7 | 1.0 | | |
| 1–7 | 13.5 | 23.3 | 0.1 | 0.4 | 0.1–1.4 |
| 8–15 | 33.7 | 25.0 | 0.3 | 0.7 | 0.2–2.6 |
| ⩾16 | 43.8 | 50.0 | 0.2 | 0.6 | 0.2–1.9 |
| Carrots | | | | | |
| 0 | 12.0 | 4.7 | 1.0 | | |
| 1–3 | 17.4 | 17.2 | 0.4 | 0.1 | 0.0–0.6 |
| 4 | 32.6 | 35.9 | 0.4 | 0.2 | 0.2–0.7 |
| ⩾5 | 38.0 | 42.2 | 0.4 | 0.2 | 0.0–0.7 |
| Cantaloupe | | | | | |
| 0 | 18.9 | 11.1 | 1.0 | | |
| 1–3 | 20.0 | 9.5 | 1.2 | 1.6 | 0.4–6.0 |
| 4 | 17.8 | 22.2 | 0.5 | 0.6 | 0.2–1.9 |
| ⩾5 | 43.3 | 57.1 | 0.4 | 0.6 | 0.3–1.7 |
| Spinach | | | | | |
| 0 | 33.3 | 27.0 | 1.0 | | |
| 1–3 | 26.9 | 49.2 | 0.4 | 0.4 | 0.2–0.9 |
| 4 | 24.7 | 12.7 | 1.6 | 1.7 | 0.6–5.0 |
| ⩾5 | 15.1 | 11.1 | 1.1 | 1.2 | 0.4-4.2 |
| Sweet potato | | | | | |
| 0 | 50.0 | 56.5 | 1.0 | | |
| 1–2 | 27.7 | 30.7 | 1.0 | 0.8 | 0.4–1.9 |
| ⩾3 | 22.3 | 12.9 | 2.0 | 2.3 | 0.8–6.4 |
| Broccoli | | | | | |
| 0 | 10.6 | 18.8 | 1.0 | | |
| 1–3 | 22.3 | 20.3 | 1.9 | 1.5 | 0.4–5.2 |
| 4 | 32.3 | 28.1 | 2.1 | 1.6 | 0.5–4.9 |
| ⩾5 | 11.8 | 32.8 | 1.8 | 1.5 | 0.5–4.7 |
| Cabbage | | | | | |
| 0 | 26.1 | 35.9 | 1.0 | | |
| 1–3 | 40.2 | 35.9 | 1.5 | 1.0 | 0.4–2.4 |
| 4 | 19.6 | 14.1 | 1.9 | 1.6 | 0.5–4.9 |
| ⩾5 | 14.1 | 14.1 | 1.4 | 1.0 | 0.3–3.2 |
| Cauliflower | | | | | |
| 0 | 26.1 | 43.8 | 1.0 | | |
| 1–3 | 40.2 | 32.8 | 1.3 | 1.4 | 0.6–3.4 |
| 4 | 19.6 | 14.1 | 3.6 | 2.9 | 1.1–8.0 |
| ⩾5 | 14.1 | 9.4 | 2.0 | 1.1 | 0.3–3.8 |
| Orange juice | | | | | |
| 0 | 11.8 | 20.3 | 1.0 | | |
| 1–29 | 36.6 | 32.8 | 1.9 | 1.2 | 0.4–3.5 |
| ⩾30 | 51.6 | 46.9 | 1.9 | 1.3 | 0.5–3.4 |
| Red meat | | | | | |
| 0 | 12.9 | 9.4 | 1.0 | | |
| 1–8 | 14.0 | 15.6 | 0.7 | 0.5 | 0.1–2.0 |
| 9–29 | 33.3 | 46.9 | 0.5 | 0.4 | 0.1–1.3 |
| ⩾30 | 39.8 | 28.1 | 1.0 | 0.7 | 0.2–2.4 |
| Vitamin A | | | | | |
| 1 (lowest quartile) | 34.0 | 25.0 | 1.0 | | |
| 2 | 16.0 | 25.0 | 0.5 | 0.3 | 0.1–0.9 |
| 3 | 31.9 | 25.0 | 0.9 | 0.7 | 0.3–2.0 |
| 4 | 18.1 | 25.0 | 0.5 | 0.5 | 0.2–1.3 |
| β-carotene | | | | | |
| 1 (lowest quartile) | 34.0 | 25.0 | 1.0 | | |
| 2 | 16.0 | 25.0 | 0.5 | 0.4 | 0.1–1.0 |
| 3 | 22.3 | 25.0 | 0.7 | 0.6 | 0.2–1.5 |
| 4 | 27.7 | 25.0 | 0.8 | 0.7 | 0.3–1.8 |
| Body mass index | | | | | |
| Lower quartile | 25.5 | 25.0 | 1.0 | | |
| Second quartile | 22.3 | 21.9 | 1.0 | 0.7 | 0.3–2.1 |
| Third quartile | 33.0 | 28.1 | 1.1 | 0.9 | 0.5–2.3 |
| Upper quartile | 19.2 | 25.0 | 0.8 | 0.7 | 0.2–1.9 |

All associations were adjusted for age, education, religion and occupation. OR, odds ratio; aOR, adjusted odds ratio.

exposures. We collected information on the spouse's occupations to determine the extent of familial asbestos exposure. None of the male subjects had spouses who worked in asbestos-related occupations. However, four of the female cases and one female control were married to men who were employed in asbestos-related jobs. When the risk estimates for the dietary variables were recalculated using personal or spousal occupation as the exposure measurement, there were small changes in the observed odds ratios for most food groups. Nevertheless, the possibility that there may be residual confounding from asbestos exposure cannot be discounted. When comparing the crude with the asbestos-adjusted odds ratios, the association with some food items changed markedly. For example, the crude and adjusted ORs for frequent cauliflower consumption ($\geqslant 5$ times per month) were 2.0 and 1.1 respectively. The ORs for tomato consumption were also attenuated after adjustment for asbestos and other factors, although fewer than 2% of controls never consumed tomatoes and therefore the adjustment must be viewed as unstable. The crude and adjusted ORs for other food items were, in general, similar.

Another consideration when interpreting these results is that all patients were interviewed at one large cancer hospital in New York City. Because there may be unusual hospital referral patterns that differ between cases and controls, there may be selection factors and biases which cannot be accounted for.

Because there has been so little research on the possible chemopreventive effects of diet and mesothelioma, the results from the Schiffman study (Shiffman *et al.*, 1988) and this study suggest that it would be worthwhile investigating further the role of diet in preventing the occurrence of mesothelioma. Such studies need to ensure that the prevalence of smoking is similar between case and control populations owing to the lower intake and distribution of some antioxidant nutrients in active smokers (Farugue *et al.*, 1995). Our findings also indicate that mesothelioma patients are leaner than control subjects, although the differences were not statistically significant.

**Acknowledgements**

The authors are grateful to Susan Harlap, Memorial Sloan-Kettering Cancer Center for her collaboration. This study was supported by NIH Grant CA-32617.

### References

AMERICAN HEALTH FOUNDATION. (1987). Proceedings of a workshop on new developments on dietary fat and fiber in carcinogenesis (optimal types and amounts of fat or fiber). *Prev. Med.*, **16**, 499–495.

BINGHAM S. (1987). The dietary assessment of individuals; methods, accuracy, new techniques and recommendations. *Nutr. Abstr. Rev.*, Series A, **57**, 705–742.

BRESLOW N AND DAY N. (1982). *Statistical Methods in Cancer Research.* Vol. 1. The design and analysis of case–control studies International Agency for Research on Cancer: Lyon.

FARUGUE MO, KHAN MR, RAHMAN MM AND AHMED F. (1995). Relationship between smoking and antioxidant nutrient status. *Br. J. Nutr.*, **73**, 625–632.

FRANCESCHI S, BIDOLI E, LA VECCHIA C, TALAMINI R, D'AVANZO B AND NEGRO E. (1994). Tomatoes and risk of digestive-tract cancers. *Int. J. Cancer*, **59**, 181–184.

KABAT CG AND WYNDER EL. (1992). Body mass index and risk of lung cancer. *Am. J. Epidemiol.*, **135**, 769–774.

KNEKT P, HELIOVAARA M, RISSANEN A, AROMAA A, SEPPANEN R, TEPPO L AND PUKKULA E. (1991). Leanness and lung-cancer risk. *Int. J. Cancer*, **49**, 208–213.

LE MARCHAND L, HANKIN JH, KOLONEL LN, BEECHER GR, WILKENS LR AND ZHAO LP. (1993). Intake of specific carotenoids and lung cancer risk. *Cancer Epidemiol. Biomarkers & Prev.*, **2**, 183–187.

MANGELS AR, HOLDEN JM, BEECHER GR, FORMAN MR AND LANZA E. (1993). Carotenoid content of fruits and vegetables: an evaluation of analytic data. *J. Am. Diet. Assoc.*, **93**, 284–296.

MUSCAT JE AND WYNDER EL. (1991). Cigarette smoking, asbestos exposure and malignant mesothelioma. *Cancer Res.*, **53**, 2263–2267.

ROSE DP, BOYAR AP AND WYNDER EL. (1986). International comparisons of mortality rates for cancer of the breast, prostate, and colon, and per capita food consumption. *Cancer*, **58**, 2363–2371.

SCHIFFMAN MH, PICKLE LW, FONTHAM E, ZAHM SH, FALK R, MELE J, CORREA P AND FRAUMENI JF Jr. (1988). Case–control study of diet and mesothelioma in Louisiana. *Cancer Res.*, **48**, 2911–2915.

SLATER TF AND BLOCK G. (eds). (1991). Antioxidant vitamins and β-carotene in disease prevention. *Am. J. Clin. Nutr.*, **53**, 189S–396S.

STEINMETZ KA, POTTER JD AND FOLSOM AR. (1993). Vegetables, fruit, and lung cancer in the Iowa Women's Health Study. *Cancer Res.*, **53**, 536–543.

TROCK B, LANZA E AND GREENWALD P. (1990). Dietary fiber, vegetables, and colon cancer: critical review and meta-analyses of the epidemiologic evidence. *J. Natl. Cancer Inst.*, **82**, 650–661.

TSUGANE S, TSUDA M, GEY F AND WATANBE S. (1992). Cross-sectional study with multiple measurements of biological markers for assessing stomach cancer risks at the population level. *Env. Health Perspect.*, **98**, 207–210.

UNITED STATES DEPARTMENT OF AGRICULTURE. (1976). *Composition of Foods, Handbook No. 8.* US Government Printing Office: Washington, DC.

WYNDER EL, HEBERT JR AND KABAT GC. (1987). Association of dietary fat and lung cancer. *J. Natl Cancer Inst.*, **79**, 631–637.